# Fear of COVID-19 in Madrid. Will patients avoid dental care?

**DOI:** 10.1016/j.identj.2021.01.013

**Published:** 2021-02-02

**Authors:** María José González-Olmo, Bendición Delgado-Ramos, Ana Raquel Ortega-Martínez, Martín Romero-Maroto, María Carrillo-Díaz

**Affiliations:** a Dentistry Department, Rey Juan Carlos University, Madrid, Spain; b Dentistry Department, Granada University, Granada, Spain; c Psychology Department, Jaén University, Jaén, Spain

**Keywords:** Anxiety, COVID-19, Coronavirus infections, Disease avoidance, Perceived fear to disease

## Abstract

**Introduction:**

The objective of this research is to describe how perceived infectability, germ aversion, and fear of COVID-19 in adults in Madrid have changed from the beginning of the pandemic until the lockdown exit phase and their influence on dental care behaviour.

**Materials and Methods:**

Some 961 participants were monitored in a study in Madrid at 2 time points: before lockdown (T0) and after completion of the total lockdown (T1). A questionnaire that included basic sociodemographic variables, the perceived vulnerability to disease scale (including perceived infectability and germ aversion), the fear of COVID-19 scale, and dental visiting behaviour after confinement for fear of COVID was administered.

**Results:**

The participants had higher scores for infectability and germ aversion at T1 than at T0 (*P* < 0.01). Of those studied, 24.5% (235) of the participants would not go to the dentist for fear of COVID-19. Those who had a high perceived infectability scale score were at least 5 times more likely to not visit the dentist. Those with high COVID-19 fear were at least 6 times more likely to not visit the dentist, and those older than 60 years were 8 times more likely to not visit.

**Conclusions:**

The population's high levels of vulnerability to infectability and perceived germ aversion associated with fear of COVID-19 and the resultant avoidance behaviour to dental care will remain until an effective drug or vaccine for SARS-CoV2 is found.

## Introduction

The epidemic coronavirus disease 2019 (COVID-19), caused by severe acute respiratory syndrome-coronavirus 2 (SARS-CoV-2), is an international public health emergency, which through the exacerbation of mental health problems such as stress, anxiety, depressive symptoms, insomnia, denial, anger, and fear raised a challenge to psychological resilience.^1^ Since the World Health Organization (WHO) officially declared the global pandemic, Madrid has established itself as one of the main foci of COVID-19 in Europe. As of May 26, Madrid was the Spanish city most affected by COVID-19. By that time, it had recorded 67,871 cases of infection, 3463 hospitalized patients, and 8977 deaths.^2^^,^^3^

The high number of patients infected with coronavirus and people who were suspected of being infected, as well as the growing number of countries affected by the outbreak, have raised concerns both nationally and globally about becoming infected. The unpredictable future of this epidemic has been exacerbated by constant media coverage and the promulgation of myths, misinformation, and the misunderstanding of health updates, which may have contributed to an increase in the fear of contagion amongst the population.^4^ The measures taken by the authorities in trying to contain the outbreak and limit contagion entailed unprecedented restrictions on mobility through social distancing and quarantine, which may have led to greater public anxiety and its immediate effects on mental health,^5^^,^^6^ possibly causing considerable psychological stress. All of this highlights the importance of emotional balance in a period of uncertainty when fear and prolonged confinement are combined.

For those who already have some kind of illness or psychological disorder, the situation can be harmful, but it may also affect others who have previously enjoyed good physical and mental health.^1^

The 2 main factors that can generate high levels of fear and anxiety are the virulence and lethality of COVID-19, especially in people older than 60 years, and those with comorbidities.^7^, ^8^, ^9^ Mental and general physical health is threatened, especially in terms of emotion and cognition.^10^ As a consequence, some people may develop a set of negative psychological responses (eg, aversion, anxiety^11^^,^^12^) and make negative cognitive assessments^13^^,^^14^ as a means of self-protection. Previous research has shown that negative emotions (eg, dental fear, anxiety, neuroticism) are associated with a lower frequency of dental visits.^15^ Dental avoidance increases the prevalence of caries^16^ and leads to a deterioration of the quality of life related to oral health.^17^

These considerations led to the general objective of analysing vulnerability to perceived infectability and germ aversion, fear of COVID-19, and the rejection of hypothetical dental clinic attendance in Spanish adults in the period from the beginning to the end of the confinement. In accordance with this, it was hypothesized that a positive association would be found between perceived vulnerability (an increase in perceived germ aversion or infectability) and fear of COVID-19 before and after confinement. In addition, we expected that significant differences would be encountered with regard to gender and COVID-19, and that perceived vulnerability before confinement may predict dental avoidance.

## Materials and methods

### Design

A repeated measures design was used with 2 time points: before lockdown (T0) and after completion of the total lockdown (T1). A self-completed questionnaire was administered to a convenience sample of adults aged 18 years and older, residing in a district of Madrid (Alcorcón), which is a representative area of the community of Madrid in terms of socioeconomic level. At T0, 1008 participants were surveyed on the streets from 1 March to 8 March 2020. At that time, the state of alarm in Spain and the confinement had not yet been declared.

The criteria for inclusion were age ≥18 years and a good understanding of the Spanish language. Each day, 3 of the researchers organized themselves into a district sampling, balancing the sample in terms of gender and age. The questionnaire was collected through a self-administered electronic format and a member of the research team was present in case any questions were raised. The nature of the study was explained, and participants were asked to give informed consent to participate in the study and, be followed up later (T1), selecting the method (WhatsApp or e-mail). Our study was approved by the Rey Juan Carlos University Ethics and Research Committee (Registration number: 0103202006520)

At T0, the survey consisted of structured questions organized into 2 sections: (i) demographic data, including age, gender, and level of education (uneducated, primary, secondary, higher education) and (ii) perceived vulnerability to disease (PVD). The questionnaire can be found in the Appendix, available online.

At T1, Spain had completed total lockdown (4-11 May 2020), and then dental clinics were allowed to reopen. All T0 participants were contacted to participate in T1. There was a 4.6% sample loss due to nonresponse at T1. Accordingly, the final sample comprised 961 participants. An online electronic questionnaire was constructed and implemented using Google Forms and included an attached consent form. The link to the questionnaire was sent by email or WhatsApp. Upon receiving and clicking on the link, participants were automatically directed to the study information and the consent form. After filling in data about the acceptance of the survey and inserting a participant code, they answered the questions that appeared sequentially. Only those who had access to the internet were allowed to participate in the study.

In this phase, the survey consisted of (i) scale of PVD (which had already been collected at T0); (ii) scale of fear of COVID-19 (published during the confinement, so it was not possible to apply it in T0); (iii) structured questions about avoidance behaviour towards the dental clinic; and (iv) a question covering whether the participant had been ill with COVID-19 (confirmation by positive polymerase chain reaction). The questionnaire can be found in the Appendix, available online.

### Instruments

PVD was assessed through the PVD scale,^18^ validated for Spanish use by Magallares et al.^19^ The PVD scale contains 15 items using a Likert scale response format that ranges from 1 (totally disagree) to 7 (completely agree). This scale has the 2 subscales of perceived infectability (7 items) and germ aversion (8 items). An example of an item in the “perceived infectability” subscale is, “I am more likely to catch an infectious disease than people in my environment.” An example of an item in the “germ-aversion” subscale is, “I prefer to wash my hands right after shaking someone's hand.” Scores were calculated by adding and averaging the 7 items of the perceived infectability subscale and the 8 items of the germ-aversion subscale. With a score range of 1 to 7, higher scores on the perceived infectability subscale reflect people's greater perceived susceptibility to infectious diseases. With a score range of 1 to 7, higher scores on the germ aversion subscale also reflect greater discomfort of individuals in situations that denote a higher probability of pathogen transmission.

The fear of COVID-19 scale (FCV-19S) was used, which was recently developed and validated by Ahorsu et al.^20^ FCV-19S was translated into Spanish using a forward- and backward-translation procedure. It contains a 7-item scale, and participants rated their agreement with the statements using a 5-point Likert scale, using the responses 1 (strongly disagree) to 5 (strongly agree) and scores in the range of 7 to 35. For instance, “It makes me uncomfortable to think about coronavirus-19.” The higher the score, the greater the patient's fear of COVID-19. The internal consistency of the FCV-19S in the present study was very good (α = 0.91).

Amongst the structured questions about avoiding the dental clinic were the following: “Are you afraid to visit the dentist for fear of COVID-19?” The response format was dichotomous (yes/no). “Are you going to the dentist?” The response format was dichotomous (yes/no).

Those who answered that they would go were asked what their reasons were for continuing to go to the dentist (“because I don't want to change my habits,” “because I have a treatment course open,” and other reasons) and were asked whether they would start aesthetic, orthodontics, or implantology treatment (yes/no).

Those who answered that they would not go were asked why (eg, fear of COVID, economic problems, or other reasons) and how long they would maintain this decision (eg, until the disease is eradicated, until I am vaccinated, until an effective medication against COVID-19 appears, or when my economy or others recover).

They were also asked independently whether they would go to the dentist in the next year for a gum problem, for a suspected cavity, or for a lost or broken filling or tooth. A 5-point Likert scale was used, which ranged from 1 (“I sure would”) to 5 (“I sure wouldn't”).

### Statistical analysis

Statistical analysis was carried out using SPSS version 24 (SPSS Inc). Data analysis included descriptive statistics and the Kolmogorov–Smirnov test to evaluate the assumption of normality, which was confirmed. Paired *t*-tests examined differences in T0-T1 for continuous variables in the sample and by gender. Pearson's correlation coefficient was used to analyse the association between continuous variables. A logistic regression analysis was carried out using attendance at a dental clinic as the dependent dichotomized variable (yes = 0, no = 1): aversion to germs in T1, perceived infectability in T1, fear of COVID in T1, being older than 60 years, and gender. The cutoff points used for the dichotomization of these variables were high aversion to germs (≥5), high aversion to infectability (≥5), and high fear of COVID-19 (≥30). The probability ratio, with a 95% confidence interval, was calculated using logistic regression analysis to evaluate dental clinic avoidance and the degree of association between avoidance and independent variables. Statistical significance was established at *P* < 0.05.

## Results

As can be seen in Table 1, the sample (*N* = 961) is composed of 402 men and 559 women, with an average age of 38.4 (±16.1) years. In terms of educational levels for the total sample, 8.7% completed primary school, 28.3% completed secondary school, and 59.6% obtained a university degree. A total of 58 participants had suffered from COVID-19, with confirmed positivity by a polymerase chain reaction test, as noted in T1.

**Table 1:** Sociodemographic characteristics in T1 by gender.

	Male (n = 402)	Female (n = 559)	Total (N = 1008)
**Age**			
M (SD)	40 (17)	37.2 (15.3)	38.4 (16.1)
18-60 years old, No. (%)	336 (35%)	501 (52.1%)	837 (87.1%)
≥60 years old, No. (%)	66 (6.9%)	58 (6%)	124 (12.9%)
**Education level**, No. (%)			
No studies	15 (1.6%)	17 (1.8%)	32 (3.3%)
Primary	49 (5.1%)	35 (3.6%)	84 (8.7%)
Secondary	113 (11.8%)	159 (16.5%)	272 (28.3%)
Higher education	225 (23.4%)	348 (36.2%)	573 (59.6%)

*N = 961.

M = mean; SD = standard deviation.

Descriptive statistics are reported in Table 2. Participants experienced significantly higher scores on T1 than T0, both in infectability (*P* < 0.01) and in aversion to germs (*P* < 0.01).

**Table 2:** Differences T0-T1 for the variables of perceived vulnerability to infection (infectability subscale and germ-aversion subscale) and fear of COVID-19.

Variables	T0 (*N* = 1008)	T1 (*N* = 961)	T0-T1 *P* value
**Vulnerability to infection**			
Infectability subscale M(SD) Score 1-3, No. (%) Score 3-5, No. (%) Score 5-7, No. (%)	3.3 (1.1) 446 (46.4%) 429 (44.6%) 86 (8.9%)	4.1 (1.1) 190 (19.8%) 516 (53.7%) 255 (26.5%)	<0.001*
Germ-aversion subscale M(SD) Score 1-3, No. (%) Score 3-5, No. (%) Score 5-7, No. (%)	3.5 (1.1) 332 (34.5%) 530 (55.2%) 99 (10.3%)	4.5 (1.1) 82 (8.5%) 548 (57%) 331 (34.4%)	<0.001*
**Fear of COVID-19** M(SD) Score 1-12, No. (%) Score 12-30, No. (%) Score 30-35, No. (%)		20.7 (6.6) 81 (8.4%) 672 (69.9%) 208 (21.6%)	

M = mean; SD = standard deviation.

⁎ Significance at the 0.01 level.

As shown in Table 3, there is a significant positive correlation between the COVID-19 fear scale and the subscales of infectability and germ aversion in T0 and in T1 (*P* < 0.01). Furthermore, a strong positive association was found between fear of COVID-19 in T1 and aversion to germs in T0 (*P* < 0.01).

**Table 3:** Cronbach's alpha and intercorrelations between subscale of infectability and germ aversion (at T0 and T1) and fear of COVID-19 (T1).

Theoretical range	Range	α	1	2	3	4	5
1. Infectability subscale T0 (1-7)	1-7	0.783		0.279*	0.587*	0.231*	0.250*
2. Infectability subscale T1 (1-7)	1-7	0.859			0.188*	0.822*	0.313*
3. Germ-aversion subscale T0 (1-7)	1-7	0.729				0.204*	0.324*
4. Germ-aversion subscale T1 (1-7)	1-6.7	0.771					0.179*
5. Fear of COVID-19 T1 (7-35)	7-35	0.913					

⁎ Correlation is significant at the .01 level.

As shown in Table 4, significant gender differences were found in the subscale of infectability (female: mean [M] = 4.2, standard deviation [SD] = 1.1; men: M = 3.9, SD = 1.1; *P* < 0.01) and germ aversion in T1 (female: M = 4.6, SD = 1.1; men: M = 4.4, SD = 1.1), with higher scores for women. Significant differences were also found for the fear of COVID-19 scale in T1 (female: M = 21.5, SD = 6.6; men: M = 19.6, SD = 6.6; *P* < 0.01), with higher scores for women. No gender differences were found in T0-T1.

**Table 4:** Variables of vulnerability to infection and fear of COVID-19 according to gender.

Variables	Man M (SD)	Woman M (SD)	Man M (SD)	Woman M (SD)	*Man* *P*	*Woman* *P*	*Man/Woman* *P*
	T0	T0	T1	T1	T0-T1	T0-T1	T0-T1
**Vulnerability to infection**							
Infectability subscale	3.2 (1.1)	3.3 (1.1)	3.9 (1.1)	4.2 (1.1)	<0.001*	<0.001*	0.891
Germ-aversion subscale	3.5 (1.1)	3.6 (1.1)	4.4 (1.1)	4.6 (1.1)	<0.001*	<0.001*	0.296
**Fear of COVID-19**			19.6 (6.6)	21.5 (6.6)			<0.001*

M = mean; SD = standard deviation.

⁎ Significance at the 0.01 level.

### Differences between participants who have been infected with COVID-19

Participants who had overcome the disease (*n* = 58) presented a higher COVID fear score (M = 23.06, SD = 6.57) than those who did not have confirmed disease (M = 20.62, SD = 6.67) (*P* < 0.01) and greater change in germ aversion in T0-T1 (M = -0.73, SD = 0.86) than the rest (M = -0.44, SD = 0.68) (*P* < 0.05).

### Avoidance of dental visit

As shown in Table 5, within the sample, 30.9% admitted to being afraid of going to the dentist because of the possibility of contagion by COVID-19 (*n* = 297), although more than half would continue to go to the dentist (*n* = 541, 56.3%). A total of 25.3% of the respondents would go to the dentist because they had not finished their treatments (*n* = 243), but 42.5% would not start aesthetic, orthodontic, or implant treatments (*n* = 408).

**Table 5:** Dentist avoidance in T1.

Are you afraid to visit the dentist for fear of COVID-19?	Yes	297 (30.9%)	No	664 (69.1%)
Are you going to the dentist in the next year?	Yes	541 (56.3%)	No	420 (43.7%)
	What are your reasons to keep going to the dentist?		Why don't you go to the dentist?	
	Treatment in progress I will not change my habits Other reasons	243 (25.3%) 250 (26%) 48 (5%)	Fear of COVID-19 Economic problems Other reasons	235 (24.5%) 154 (16%) 31 (3.2%)
	Would you start an aesthetic treatment, orthodontic or implant treatment?		How long would you keep this decision?	
	Yes No	133 (13.8%) 408 (42.5%)	Until the disease is eradicated Until I am vaccinated Until an effective medication against COVID-19 appears When my economy or others recover	110 (11.4%) 70 (7.3%) 115 (12%) 125 (13%)
Would you go to the dentist in the next year for a gum problem?	Yes	762 (79.3%)	No	199 (20.7%)
Would you go to the dentist in the next year for a suspected cavity?	Yes	767 (79.8%)	No	194 (20.2%)
Would you go to the dentist in the next year for a lost or broken filling or tooth?	Yes	804 (83.7%)	No	157 (16.3%)

Of the total sample, 43.7% would not go to the dentist (*n* = 420), 24.5% for fear of COVID-19 (*n* = 235), 16% because of financial problems (*n* = 154), and 3.2% for others reasons (*n* = 31). More than half of the respondents would maintain this decision until the disease is eradicated or an effective treatment is found. In addition, 20.7% (*n* = 199) would not go to the dentist even if they had gum problems; 20.2% would not go even if they suspected that they might have cavities (*n* = 194); and, lastly, 16.3% would not go even if fillings or teeth were fractured (*n* = 157).

A logistic regression analysis was carried out with the Hosmer–Lemeshow test. The logistic model is considered adequate (0.732) and explains 35.2% of the variability from Nagelkerke's R-square value (Table 6).

**Table 6:** Results of the logistic regression model for dentist avoidance.

Variables	OR	CI (95%)	*P*
High perceived Infectability	4.21	2.87-5.64	<0.001*
High fear of COVID-19	5.18	2.96-9.4	<0.001*
Older than 60 years old	7.63	3.56-15.35	<0.001*

CI = confidence interval; OR = odds ratio.

⁎ Significance at the .01 level.

Based on multivariate analysis, the 3 variables that showed a significant relationship with *P* = .001 were perceived infectability, fear of COVID-19, and being older than 60 years. The respondents who had a high score on the scale of perceived infectability were at least 5 times more likely not to visit the dentist (odds ratio [OR] = 4.21, β = 1.43). Those with a COVID-19 fear score above 30 were at least 6 times more likely not to visit the dentist (OR = 5.18, β = 1.64). Finally, participants older than 60 were 8 times more likely not to go to the dental clinic (OR = 7.63, β = 2.03).

## Discussion

The results obtained in this study may clarify the increased levels of vulnerability to infectability and germ aversion in the Spanish population promoted by the fear of COVID-19 during a 2-month pandemic period (March and April 2020). Research on other infectious disease outbreaks suggests that individual difference variables, such as PVD, may play a role in coronavirus phobia and the development of xenophobia or social discrimination related to the said virus.^21^

Furthermore, a large percentage of citizens say they would not go to a dental surgery other than for an emergency until effective treatment for COVID-19 or a vaccine is found. Such avoidance behaviour is typified by Hayes et al and promoted by fear of contracting the virus.^22^ In previous studies, the prevalence of fear of COVID-19 has not been specified as other emotional states predictive of fear, such as anxiety have. Specifically, the prevalence of anxiety after confinement varies from 27.2% to 38.7% in different studies.^23^^,^^24^

The results of our study also reveal a significant difference in terms of fear of infection, with this fear being greater in women than in men. This difference may be due to women knowing how to recognize and express their feelings and uncertainty better than men.^25^ Recent COVID-19 studies have also endorsed the fact that gender is a consistent predictor of negative affective states such as anxiety, stress, or depression,^26^ as well as showing that women are more careful in implementing hygiene measures than men are in general. Therefore, in a pandemic situation, they are more aware of the risk of COVID-19 disease from failure to comply with appropriate hygiene measures.^27^^,^^28^

The data show that 43.7% of those surveyed would not seek dental services, and of that percentage, 33.8% would not go for fear of contagion from COVID-19 and 44.3% because of economic problems arising from the pandemic. Previous studies support the serious impact on economic life caused by the pandemic.^29^^,^^30^ The World Trade Organization (WTO) and the Organisation for Economic Co-operation and Development (OECD) identified the COVID-19 pandemic as the greatest threat to the economy since the financial emergency of 2008-2009. Some experts have even said that the world is facing the greatest emergency since World War II. It is estimated that there will be an approximate monthly loss of 2% in annual gross domestic product (GDP) growth.^29^ Other authors report that rampant unemployment best describes this crisis with consequences for the psychological, economic, and social well-being of individuals.^30^

The limitations of this study are linked to the sample used, which has an associated bias in that it is not random, which may have influenced the results and limited the generalisation of the findings to a broader population, although the gender of the participants was chosen in an equitable manner. A possible second limitation comes from the use of self-reporting measures, which may be affected by responses based more on social desirability than reality. Finally, the COVID-19 fear discussion would have methodological limitations because the COVID-19 measurement instrument was validated and published after the commencement of our study.^31^

This research has some implications that may be of some relevance to dental practice. It is quite possible that the presence of COVID-19 amongst the public has led to a certain degree of rejection of dental services. Psychological support will be needed to assist patients in the face of emotional disturbances, some of which may be linked to the pandemic and to help them overcome levels of fear and anxiety.^32^ Exposure therapy may be an effective resource in improving certain avoidance behaviour.^33^ In addition, since chronic stress is an important modulator of immunity, it may be linked to and thus directly influence the likelihood of infection.

It is possible that after this pandemic, due to the increased fear of COVID-19, avoidance behaviour will develop in dental practices and also in other medical specialties. It is important to identify the people at risk of developing negative emotions and avoidance tendencies to try to reduce the impact on the population's overall health.

Furthermore, the prevention-based approach to dentistry may be negatively influenced by this new era of COVID-19 fear, which may bring the patient to the dental clinic only for urgent or curative treatments and discard the preventive support so important for oral health. Specialists recommend that patients be examined every 6 months or at least once a year.

Early intervention can help to avoid invasive treatments such as tooth extraction. Regular consultation spares patients from exposure to pain and oral pathology, as well as from the side effects associated with the spread of infections.^34^, ^35^, ^36^, ^37^, ^38^ In summary, promotion, prevention, and education at the individual and general population level should focus on psychosocial support for the management of the effects derived from the fear of the virus, as well as on emphasizing messaging focusing on the importance of periodic consultations for the maintenance of oral and general health.^35^^,^^36^^,^^39^, ^40^, ^41^

Future lines of research will be necessary to assess to what extent the fear of COVID-19, the perception of vulnerability, and the population's aversion to germs could be associated with difficulties related to causing problems in oral, systemic, and mental health or if the passage of time will allow people to become familiar with the presence of the virus, mitigating current rejection behaviours.

## Conclusion

Our study shows the population's high level of vulnerability to infectability and perceived germ aversion as being possibly associated with COVID-19 and that the resultant avoidance behaviour to dental care will remain until an effective drug or vaccine for SARS-CoV2 is found.

## Appendix Supplementary materials

Image, application 1

## Footnotes

Supplementary material associated with this article can be found, in the online version, at doi:10.1016/j.identj.2021.01.013.
