# Supplementary material for: Fear of COVID-19 in Madrid. Will patients avoid dental care?
Source: Int Dent J. 2021 Feb 2;72(1):76–82. doi: 10.1016/j.identj.2021.01.013 (PMC7970159; doi:10.1016/j.identj.2021.01.013)
Supplement: Supplementary file 1 [file mmc1.pdf]

T0 Questionnaire

*Mandatory

1. Insert a code (please, enter your last two digits of your identity card, then the initial of your first name followed by the initial of your last name) *

2. Age *

3. Gender *

- Male
- Female

4. Level of education *

- Uneducated
- Primary
- Secondary
- University degree

5. Have you suffered from COVID-19 confirmed by a PCR test? *

- [ ] Yes
- [ ] No

Are you agree with the following statements?:

6. It really bothers me when people sneeze without covering their mouths *

	1	2	3	4	5	6	7	
Totallydisagree	◯	◯	◯	◯	◯	◯	◯	Completely agree

7. If an illness is ‘going around’, I will get it *

	1	2	3	4	5	6	7	
Totallydisagree	◯	◯	◯	◯	◯	◯	◯	Completely agree

8. I am comfortable sharing a water bottle with a friend *

	1	2	3	4	5	6	7	
Totallydisagree	◯	◯	◯	◯	◯	◯	◯	Completely agree

9. I do not like to write with a pencil someone else has obviously chewed on *

	1	2	3	4	5	6	7	
Totallydisagree								Completely agree

10. My past experiences make me believe I am not likely to get sick even when my friends are sick *

	1	2	3	4	5	6	7	
Totallydisagree								Completely agree

11. I have a history of susceptibility to infectious disease *

	1	2	3	4	5	6	7	
Totallydisagree								Completely agree

12. I prefer to wash my hands pretty soon after shaking someone's hand *

	1	2	3	4	5	6	7	
Totallydisagree								Completely agree

13. In general, I am very susceptible to colds, flu and other infectious diseases *

	1	2	3	4	5	6	7	
Totallydisagree								Completely agree

14. I dislike wearing used clothes because you do not know what the last person who wore it was like *

	1	2	3	4	5	6	7	
Totallydisagree								Completely agree

15. I am more likely than the people around me to catch an infectious disease *

	1	2	3	4	5	6	7	
Totallydisagree								Completely agree

16. My hands do not feel dirty after touching money *

	1	2	3	4	5	6	7	
Totallydisagree								Completely agree

17. I am unlikely to catch a cold, flu or other illness, even if it is 'going around *

	1	2	3	4	5	6	7	
Totallydisagree								Completely agree

18. It does not make me anxious to be around sick people *

	1	2	3	4	5	6	7	
Totallydisagree								Completely agree

19. My immune system protects me from most illnesses that other people get *

	1	2	3	4	5	6	7	
Totallydisagree								Completely agree

20. I avoid using public telephones because of the risk that I may catch something from the previous user *

	1	2	3	4	5	6	7	
Totallydisagree								Completely agree

T1 Questionnaire

*Mandatory

1. Insert a code (please, enter your last two digits of your identity card, then the initial of your first name followed by the initial of your last name) *

2. Have you suffered from COVID-19 confirmed by a PCR test? *

   - Yes
   - No

Are you agree with the following statements?:

3. It really bothers me when people sneeze without covering their mouths *

	1	2	3	4	5	6	7	
Totally disagree								Completely agree

4. If an illness is ‘going around’, I will get it *

	1	2	3	4	5	6	7	
Totally disagree								Completely agree

5. I am comfortable sharing a water bottle with a friend *

	1	2	3	4	5	6	7	
Totally disagree								Completely agree

6. I do not like to write with a pencil someone else has obviously chewed on *

	1	2	3	4	5	6	7	
Totally disagree								Completely agree

7. My past experiences make me believe I am not likely to get sick even when my friends are sick *

	1	2	3	4	5	6	7	
Totally disagree								Completely agree

8. I have a history of susceptibility to infectious disease *

	1	2	3	4	5	6	7	
Totally disagree								Completely agree

9. I prefer to wash my hands pretty soon after shaking someone’s hand *

	1	2	3	4	5	6	7	
Totally disagree								Completely agree

10. In general, I am very susceptible to colds, flu and other infectious diseases *

	1	2	3	4	5	6	7	
Totally disagree								Completely agree

11. I dislike wearing used clothes because you do not know what the last person who wore it was like *

	1	2	3	4	5	6	7	
Totally disagree								Completely agree

12. I am more likely than the people around me to catch an infectious disease *

	1	2	3	4	5	6	7	
Totally disagree								Completely agree

13. My hands do not feel dirty after touching money *

	1	2	3	4	5	6	7	
Totally disagree								Completely agree

14. I am unlikely to catch a cold, flu or other illness, even if it is 'going around *

	1	2	3	4	5	6	7	
Totally disagree								Completely agree

15. It does not make me anxious to be around sick people *

	1	2	3	4	5	6	7	
Totally disagree								Completely agree

16. My immune system protects me from most illnesses that other people get *

	1	2	3	4	5	6	7	
Totally disagree								Completely agree

17. I avoid using public telephones because of the risk that I may catch something from the previous user *

	1	2	3	4	5	6	7	
Totally disagree								Completely agree

Please answer the following questions honestly:

18. I am most afraid of coronavirus-19 *

	1	2	3	4	5	
Strongly disagree						Strongly agree

19. It makes me uncomfortable to think about coronavirus-19 *

	1	2	3	4	5	
Strongly disagree						Strongly agree

20. My hands become clammy when I think about coronavirus-19 *

	1	2	3	4	5	
Strongly disagree	○	○	○	○	○	Strongly agree

21. I am afraid of losing my life because of coronavirus-19 *

	1	2	3	4	5	
Strongly disagree	○	○	○	○	○	Strongly agree

22. When watching news and stories about coronavirus-19 on social media, I become nervous or anxious *

	1	2	3	4	5	
Strongly disagree	○	○	○	○	○	Strongly agree

23. I cannot sleep because I'm worrying about getting coronavirus-19 *

	1	2	3	4	5	
Strongly disagree	○	○	○	○	○	Strongly agree

24. My heart races or palpitates when I think about getting coronavirus-19 *

	1	2	3	4	5	
Strongly disagree	○	○	○	○	○	Strongly agree

25. Are you afraid to visit the dentist for fear of COVID-19? *

- ○ Yes
- ○ No

26. Are you going to the dentist in the next year? *

- ○ Yes
- ○ No

Participants who answered "No", continued the form on question number 29.
Participants who answered "Yes", continued filling the form normally excepting the questions 29 and 30.

27. What are your reasons to keep going to the dentist? * Only participants who answered "Yes" in 26.

- ○ Because I don't want to change my habits
- ○ Because I have a treatment course open
- ○ Other reasons

28. Would you start an aesthetic treatment, orthodontic or implant treatment? * Only participants who answered “Yes” in 26.

- Yes
- No

29. Why don't you go to the dentist? * Only participants who answered “No” in 26.

- Fear of COVID-19
- Economic problems
- Other reasons

30. How long would you keep this decision? * Only participants who answered “No” in 26.

- Until the disease is eradicated
- Until I am vaccinated
- Until an effective medication against COVID-19 appears
- When my economy or others recover

31. Would you go to the dentist in the next year for a gum problem? *

	1	2	3	4	5	
I sure would						I sure wouldn´t

32. Would you go to the dentist in the next year for a suspected cavity? *

	1	2	3	4	5	
I sure would						I sure wouldn´t

33. Would you go to the dentist in the next year for a lost or broken filling or tooth? *

.

	1	2	3	4	5	
I sure would	◯	◯	◯	◯	◯	I sure wouldn´t
